# Supplementary material for: Botryocladia leptopoda Extracts Promote Wound Healing Ability via Antioxidant and Anti-Inflammatory Activities and Regulation of MMP/TIMP Expression
Source: Mar Drugs. 2025 Nov 19;23(11):444. doi: 10.3390/md23110444 (PMC12653739; doi:10.3390/md23110444)
Supplement: Supplementary file 1 [file marinedrugs-23-00444-s001.zip › marinedrugs-3961942-supplementary.pdf]

Supplementary data

(a)

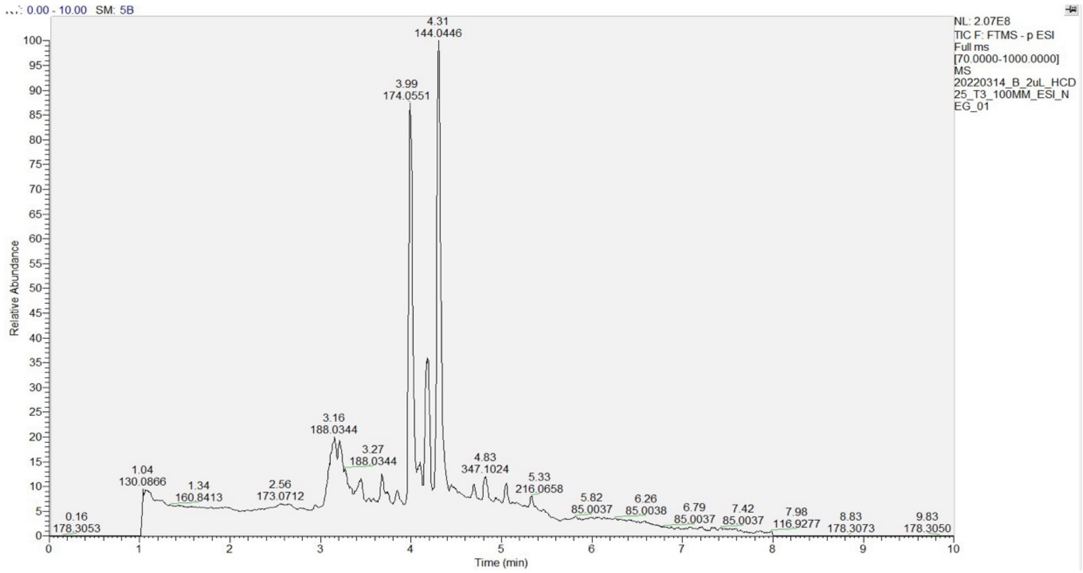

(b)

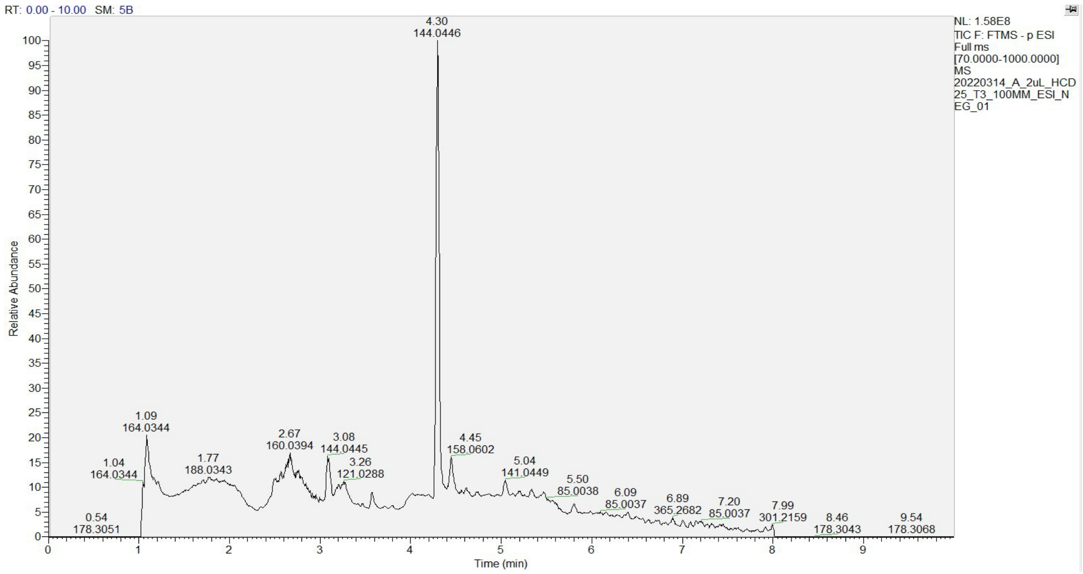

Figure S1. The profile of *B. leptopoda* extracts. (A) AE extraction and (B) FE extraction.
